# Potential Effects of Microplastic on Arbuscular Mycorrhizal Fungi

**DOI:** 10.3389/fpls.2021.626709

**Published:** 2021-02-01

**Authors:** Eva F. Leifheit, Anika Lehmann, Matthias C. Rillig

**Affiliations:** ^1^Institut für Biologie, Freie Universität Berlin, Berlin, Germany; ^2^Berlin-Brandenburg Institute of Advanced Biodiversity Research, Berlin, Germany

**Keywords:** microplastic, global change, earth system processes, pollution, arbuscular mycorrhizal fungi

## Abstract

Microplastics (MPs) are ubiquitously found in terrestrial ecosystems and are increasingly recognized as a factor of global change (GCF). Current research shows that MP can alter plant growth, soil inherent properties, and the composition and activity of microbial communities. However, knowledge about how microplastic affects arbuscular mycorrhizal fungi (AMF) is scarce. For plants it has been shown that microplastic can both increase and decrease the aboveground biomass and reduce the root diameter, which could indirectly cause a change in AMF abundance and activity. One of the main direct effects of microplastic is the reduction of the soil bulk density, which translates to an altered soil pore structure and water transport. Moreover, especially fibers can have considerable impacts on soil structure, namely the size distribution and stability of soil aggregates. Therefore, microplastic alters a number of soil parameters that determine habitat space and conditions for AMF. We expect that this will influence functions mediated by AMF, such as soil aggregation, water and nutrient transport. We discuss how the impacts of microplastic on AMF could alter how plants deal with other GCFs in the context of sustainable food production. The co-occurrence of several GCFs, e.g., elevated temperature, drought, pesticides, and microplastic could modify the impact of microplastic on AMF. Furthermore, the ubiquitous presence of microplastic also relates to earth system processes, e.g., net primary production (NPP), carbon and nitrogen cycling, which involve AMF as key soil organisms. For future research, we outline which experiments should be prioritized.

## Introduction

Microplastics (MPs) are ubiquitously found around the globe and are increasingly recognized as a factor of global change (GCF; Rillig and Lehmann, 2020). While MP research has focused on marine and freshwater ecosystems for a long time, recently attention has shifted to terrestrial ecosystems. MP is expected to enter soil ecosystems predominantly in agricultural fields through fertilization with sewage sludge and compost (Nizzetto et al., 2016; van den Berg et al., 2020). But MP emissions also reach soils *via* atmospheric deposition, runoff or aerial transport from nearby roads, where particles and fragments are generated through tire and road wear abrasion or from sports grounds with artificial substrates, through the addition of polymer coated fertilizer, littering, flooding events, or irrigation with wastewater (Piehl et al., 2018; Bergmann et al., 2019; Baensch-Baltruschat et al., 2020; Brahney et al., 2020). However, direct analysis of the concentration of MP in the soil is problematic due to analytical difficulties. MP is quickly incorporated into the soil matrix where it “disappears,” and thus cannot be easily distinguished from the soil organic matter without dedicated extraction and analytical protocols.

Arbuscular mycorrhizal fungi (AMF) are a key member of terrestrial ecosystems: by entangling soil aggregates with their hyphae, they improve soil structure and potentially stabilize carbon in soil aggregates (Rillig and Mummey, 2006; Verbruggen et al., 2016). They ramify throughout the soil to access nutrients, which they supply to their symbiotic host partner (Parniske, 2008). In exchange for these nutrients, plants provide carbohydrates and fatty acids to the fungus inside root cells where the fungus forms arbuscules for this exchange (Parniske, 2008; Keymer et al., 2017). Arbuscules or other structures such as hyphae and vesicles inside the roots are intraradical measures for activity and abundance, whereas soil mycelial length, spore numbers, and phosphatase activity are used as extraradical abundance and activity measures, which are complemented by molecular techniques such as qPCR (Thonar et al., 2012). As ubiquitous members of the soil microbial community, AMF face a variety of adverse conditions and likely multiple co-occurring GCFs, including novel pollution agents such as microplastic.

Most plastic types are persistent in the environment and are expected to accumulate in the soil, which likely leads to continuously increasing concentrations of MPs in soils (Rillig, 2012). Current research shows that microplastic can alter plant performance, soil properties, and the composition and activity of microbial communities (e.g., Machado et al., 2018; Boots et al., 2019). Some of the soil biota, e.g., nematodes and Rotifera, can be very sensitive to MP and show alterations of the gut microbiome, reproduction rate, motility and life span, show stress reactions, and malfunctioning metabolism in response to different types of MP (Büks et al., 2020). Effects on AMF can equally be expected, but specific knowledge on MP impacts on AMF is scarce. We expect a number of direct effects of MP on AMF, such as the toxicity of some plastic components (additives), as well as indirect effects of MP on AMF *via* altered plant performance and soil properties. We elaborate these effects in the following sections, followed by a discussion on potential interactive effects of MP with other GCFs and feedbacks from earth system processes and we finish with an outline of future research priorities.

## Hypothesized Direct Effects of Microplastic on AMF

Microplastic can have direct harmful effects on soil biota as they contain a variety of additives that can be toxic (Kim et al., 2020). The authors of this study demonstrated that the acute toxicity of the MP on nematodes disappeared when the additives were extracted before exposure. Additionally, there is an increasing body of literature showing that organic pollutants (e.g., polycyclic aromatic hydrocarbons or organochlorines such as DDT), polychlorinated biphenyls, antibiotics, herbicides, pesticides, and trace metals can absorb to plastic surfaces (Wang et al., 2019). Especially small MP particles or high concentrations can induce stress reactions, alter metabolic processes, reproduction, and mortality (Büks et al., 2020). MP can directly alter the composition of the bacterial community in soil and for aquatic systems this has been shown for fungal communities (Kettner et al., 2017; Fei et al., 2020). AMF, like other soil biota, can be negatively affected by pollutants, e.g., heavy metals or hydrocarbons (Cabello, 1997; Joner and Leyval, 2003; Wang et al., 2020). We thus expect direct effects of the MP additives or the pollutants absorbed on the MP surface, which will eventually be released upon degradation. Although AMF exhibit a certain tolerance to heavy metals and hydrocarbons, and can even assist in reducing their toxicity to plants, they are negatively affected at high concentrations (Cabello, 1997; Ferrol et al., 2016). Typical reactions of AMF to soil pollutants include reduced root colonization and infectivity, reduced arbuscule and spore numbers or cell damage (Cabello, 1997; Leyval et al., 1997; Desalme et al., 2012; Ferrol et al., 2016). Pharmaceuticals like antibiotics can exhibit mycotoxicity for AMF as well, reducing hyphal length and spore numbers (Hillis et al., 2008). Additionally, changes in AMF community structure can occur with soil pollution: e.g., in soils with high lead contamination the abundance of *Acaulosporaceae* and *Glomeraceae* decreased, while the relative abundance of *Paraglomeraceae* increased (Faggioli et al., 2019). The binding of (toxic) pollutants to persistent MP would lead to their accumulation in the soil and over time, the pollution load for AMF will be further augmented, considering the projected increases in use of plastic, antibiotics, and other human activities that release pollutants (Tilman et al., 2002; Lebreton and Andrady, 2019; Roberts and Zembower, 2020). At the moment, it is difficult to estimate a realistic time scale for MP degradation and release of components, because there is not enough information on degradation processes in the soil and the diversity of plastic types and sizes is too large.

Microplastics can furthermore directly influence AMF *via* breakdown products. Especially biodegradable plastics produce dodecanal, which can be accumulated in the rhizosphere and is known to negatively affect plant and fungal growth (Qi et al., 2020). One recent study showed a strong change in AMF community composition and diversity under MP pollution (Wang et al., 2020). The authors found that the relative abundance of AMF taxa depended on type and concentration of MP: e.g., *Glomeraceae* were reduced in treatments with biodegradable polylactic acid (PLA) compared to the control and treatments with polyethylene (PE); OTU numbers of *Ambispora* and *Archaeosporaceae* increased at higher application rates of MP (10% addition compared to 1% addition) under PLA and PE for *Ambispora* and only under PLA for *Archaeosporaceae*. AMF diversity was found to be highest under 10% PLA addition and, overall, the biodegradable PLA had a larger influence on AMF diversity compared to PE.

## Hypothesized Indirect Effects of Microplastic on Plant Host, Extraradical Soil Environment and Microbial Communities

The largest influence of MPs on soil is the change in bulk density, which often has positive consequences for plant growth as this reduces the root penetration resistance, improves the water holding capacity (WHC; see Machado et al., 2019) and is often accompanied by a better aeration (Niu et al., 2012). These conditions favor nutrient and water supply to plants, leading to increased root and shoot biomass, but also to altered root traits (Materechera et al., 1992; Machado et al., 2019; Rillig et al., 2019b). This has been demonstrated in recent studies, where MP reduced the root diameter or increased the fine root length (Machado et al., 2019; Lehmann et al., 2020a). However, from a mere change in diameter or length of fine roots, conclusions on AMF reactions cannot be drawn as there are important functional differences within fine roots: First to third order fine roots are the absorptive part of the root system, where most of the AMF structures are usually found, whereas higher order fine roots are more active in transport and less likely to host AMF (McCormack et al., 2015). Additionally, thin diameter fine roots have a higher absorptive surface area compared to thicker fine roots and can show lower colonization rates by AMF because they do not need to rely on a mycorrhizal partner for nutrient uptake (Eissenstat et al., 2015). Fine root traits differ substantially between plant types, e.g., the average of absorptive fine roots is 33% for woody plants and 81% for herbaceous plants (McCormack et al., 2015). Although general predictions of the effect of altered root traits on AMF colonization is difficult with the current literature, it is fair to assume that altered root traits will likely lead to changes in intraradical and extraradical fungal traits, such as root colonization, hyphal extension, abundance, and branching behavior (Rillig et al., 2015; Cockerton et al., 2020). Two studies have found an increase in root colonization by 8 and 22%, respectively, with polyester fiber addition, supporting this postulation (Machado et al., 2019; Lehmann et al., 2020a). However, other plastic types (PA, PEHD, PET, PP, and PS) have not induced an increase in colonization (Machado et al., 2019). Therefore, the effect predominantly depends on the MP parameters (concentration, type, shape, and additives), but also on the specific host-symbiont-relationship: A mycorrhizal symbiosis with a highly dependent plant host might be more strongly influenced by MP-induced alterations compared to a symbiosis in which the plant host is not as dependent on AMF; mycorrhizal dependent plants might profit more from increased AMF activity, i.e., receive more nutrients or water, supporting plant growth. Additionally, there will certainly be differences in the reactions to MP between single AMF species, i.e., some species might be more susceptible to adverse soil conditions (see section “Hypothesized direct effects of microplastic on AMF”).

In some cases, MP has reduced plant growth (Qi et al., 2018; Wang et al., 2020), which could limit the C allocation to AMF, and thus reduce AMF abundance and activity, including the supply of nutrients and water to the plant.

Microplastic additions to soil clearly influence soil structure: Laboratory studies found positive and negative effects, but reductions of aggregate stability and aggregate size have been observed more frequently (Lehmann et al., 2020b). Especially fibers with their linear shape can reduce soil aggregate stability and mean weight diameter (by currently unknown mechanisms; Lehmann et al., 2020b), and thus pore size distribution. Smaller pores and improved oxygen availability could change hyphal ramification (Crawford, 1992; Drew et al., 2003). In relation with a reduced bulk density of the soil through MP addition it can be expected that hyphae, like roots, experience a reduced penetration resistance and will thus be able to explore more soil space. This assumption is supported by several studies that show increased root growth due to decreased soil bulk density, leading to improved root colonization with AMF, which facilitated nutrient uptake by the plants (Nadian et al., 1996; Entry et al., 2002). Assuming a reduced bulk density and smaller mean weight diameter of soil aggregates, associated with smaller soil pores, changes in water transport and WHC are expected. Soil water is usually only available to plants in soil pores >5μm (Weil and Brady, 2017). These smaller soil pores can easily be penetrated by AM hyphae, which can have diameters as small as 1.2μm (Dodd et al., 2012). The creation of more smaller pores by MP could thus foster the AMF assisted water supply to plants.

Another soil property that can be changed by MP is the soil pH. Depending on polymer type and chemistry, pH can be increased or decreased: in a recent study non-biodegradable PE decreased the soil pH, while biodegradable PLA increased the soil pH and such effects on soil pH can affect AMF community composition (Wang et al., 2020); however, in another study, high density polyethylene decreased the soil pH but PLA had no effect (Boots et al., 2019). Soil pH plays a crucial role for the composition of microbial communities in general and of AMF communities in particular (Porter et al., 1987; Aciego Pietri and Brookes, 2009). Thus, Wang et al. (2020) suggest that the observed effects of MP on the AMF community are mediated by soil pH.

Microplastic additions to soil have varying effects on the overall microbial community composition and activity, likely as a function of concentration and chemical composition of MP (Liu et al., 2017; Machado et al., 2018, 2019; Yu et al., 2020). The underlying mechanisms can be assumed to be in the change of soil properties, especially bulk density, which improves the aeration of the soil and could thus stimulate aerobic microorganisms; or more generally speaking MP induces a shift in the microbial community composition (Liu et al., 2017). Ren et al. (2020) studied effects of two different MP particle sizes (<150 and <13μm) on microbial communities and found increases and decreases of richness and diversity, depending on MP particle size, with smaller particles tending to increase these parameters. When MP was added to the soil, the microbial community structure changed, e.g., *Actinobacteria* increased in soils with MP, whereas other groups such as *Proteobacteria* or *Acidobacteria*, and for smaller particles also some fungal groups, e.g., *Basidiomycota* and *Chytridiomycota* were reduced in MP treatments (Ren et al., 2020). Similar observations, i.e., dominating *Actinobacteria* and reduced *Proteobacteria*, have been made by other authors (Huang et al., 2019; Zhang et al., 2019). The soil microbial community composition can have a strong influence on AMF: for example, there are “mycorrhiza helper bacteria” (e.g., *Pseudomonas* sp., *Burkholderia* sp.) that facilitate root colonization or hyphal growth from spores (Frey-Klett et al., 2007; Viollet et al., 2017) and nitrogen-fixing bacteria can help AMF to maximize nutrient acquisition in the host (van der Heijden et al., 2016). *Proteobacteria*, which can be reduced with MP pollution (Ren et al., 2020), can have interactions with AMF: their presence can alter the structure of AMF assemblages and in contaminated soils it can increase root colonization (Dagher et al., 2020).

In addition to microbial community composition the microbial activity can be altered by MP (Liang et al., 2019). One of the mechanisms that can be responsible for this alteration is the addition of an energy resource through MP, as MP itself represents organic carbon (Rillig, 2018). Although generally inert, MPs are mainly composed of carbon, which can partly leach as dissolved organic carbon before fragmentation occurs (Romera-Castillo et al., 2018). The degradation of thermoplastics in soil varies among plastic types and largely depends on the presence of UV-light (Scalenghe, 2018; Chamas et al., 2020). Elastomers such as tire particles can have a rather short half-life of only 16months (Baensch-Baltruschat et al., 2020). This introduces an artificial resource into the soil that potentially changes the activity of the natural microbial community. This effect has been observed in several studies: e.g., Machado et al. (2019) found an increase in fluorescein diacetate hydrolase (FDA) by several MP types (polyamid beads, polyester fibers, and pellets of high density polyethylene); Liu et al. (2017) found an increase in FDA and phenol oxidase by polypropylene-microparticles. In a plastic-free soil, it could be shown that there are strong synergistic interactions between *Rhizophagus irregularis* and other soil microbes: more than half of the N that the AMF provided to the plant could be related to a synergistic interaction with the host and the soil microbial community (Hestrin et al., 2019). The MP-induced increase in microbial activity could have an indirect effect on AMF: it could trigger similar synergies between AMF and other soil microbes, leading to increased nutrient uptake by plants.

On the other hand, there are studies showing negative effects of MP on enzyme activities, e.g., polyethylene mulching film reduced catalase, laccase, and phenol oxidase (Yu et al., 2020). Differences among studies can be explained by use of different MP types, concentrations, and experiment durations. Some MP types might have toxic effects (additives, absorbed pollutants, see section “Hypothesized direct effects of microplastic on AMF”) or alter the soil properties in a way which is disadvantageous for some species, thus reducing overall activity and enzyme production (Yu et al., 2020). These MP-induced reduced microbial activities could also have an indirect effect on AMF: it could prevent synergistic interactions between AMF and other microbes or reduce positive interactions between AMF and “mycorrhiza helper bacteria” if their activity is decreased as a consequence to MP pollution.

## Effects of Microplastic on AMF in a Perspective of Global Change

Long-term consequences of MP effects cannot currently be foreseen, as effects are only studied in the short-term and there are no realistic estimates of MP accumulation in soils. But long-term predictions are necessary as deposited MP will interact with other environmental impacts in the future. In fact, MP is increasingly recognized as a GCF (Rillig and Lehmann, 2020) and should thus be studied in the context of other GCFs, instead of being regarded in isolation.

As MP is now found in almost every ecosystem around the globe, multiple GCFs will likely occur in combination. The co-occurrence of several GCFs could intensify the impact of each single factor (Rillig et al., 2019d). For grassland ecosystems, in which the AM symbiosis is the dominant mycorrhizal type, the occurrence of GCFs such as elevated temperature, elevated CO_2_, drought, pesticides, heavy metals, and MP is likely. MP research in general has been heavily focused on agricultural systems; this means, we know little about effects in other ecosystem types (such as tropical forests or Mediterranean woodlands), and the same applies to effects on AMF. Elevated CO_2_ has been shown to stimulate AMF activity (Sanders et al., 1998; Drigo et al., 2010), like MP did for AMF colonization (Machado et al., 2019; Lehmann et al., 2020a). AMF have repeatedly been proposed as an important contributor for sustainable agriculture, where they contribute to resistance and resilience against GCFs such as drought, salinity, but also against pathogens (Veresoglou and Rillig, 2012; Rillig et al., 2016, 2019a; Begum et al., 2019). This role, however, can be challenged if AMF are affected by MP. If MP alters soil porosity and water transport of the soil, AMF-mediated supply with water and nutrients to plants could be assigned a different relevance in both directions. In a recent study, it could not be shown that AMF helped mitigate negative effects of the GCFs MP and drought (Lehmann et al., 2020a), which would represent a diminished role for AMF in ecosystem resilience. It is possible that a negative impact of MP prevented the plant protection that AMF usually provide. If AMF are directly affected by MP, e.g., its toxic components, their ability to protect plants from pathogens or co-occurring GCFs could be changed. We think that this is an important question highlighting the role of AMF for sustainable agriculture in times of global change and for the production of healthy, nutritional food.

Agricultural soils are among the most strongly MP-polluted terrestrial ecosystems because they receive organic fertilizers (e.g., compost, sewage sludge, and biowaste fermentation digestates) that can contain high numbers of MP particles (Mahon et al., 2017; Weithmann et al., 2018) and additional sources of MP such as fragments from mulching films that can be extensive (Zhang et al., 2019). Many crops form AM-symbioses. The association of a crop-host with AMF can have positive effects on nutrient uptake and biomass yield, can enhance drought-, metal-, and salinity-tolerance, reduce nutrient leaching, improve soil structure, and increase plant biodiversity (Thirkell et al., 2017). AMF have been proposed to assist in stabilizing sustainable forest and agricultural productivity in the struggle with increasing impact of GCFs (Solaiman et al., 2014; Sosa-Hernández et al., 2019), which now include MPs. However, the colonization by AMF can also reduce yields, depending on the crop species identity and soil nutrient status (Hoeksema et al., 2010). Many of the well-recognized AMF functions (especially improvement of soil structure and N retention) are connected to key ecosystem services that are important for soil health and eventually for human health, as humans depend on the production of healthy food from soils and the filtration of water for potable groundwater (Lehmann et al., 2020c). MP has the potential to interfere with these important ecosystem functions, as outlined in the sections above, consequently affecting nutrient cycling, i.e., nutrient supply and release of N, with spillover effects on net primary production (NPP). AMF often increase the nutrient status of crops, thereby improving food quality (Thirkell et al., 2017). MP often increases plant growth, but if this positive effect is supported by increased nutrient uptake through an association with AMF is currently not known.

The current state-of-the-art only shows us pieces of a larger puzzle, in which we know that AMF, MP, and other GCFs all contribute to soil and plant biodiversity (Lozano and Rillig, 2020), which are also key for soil (and human) health. But how these factors interact and what the outcome of their interactions are, is difficult to predict (Rillig et al., 2019d).

## Potential Earth System Feedbacks and the Role of AMF

Accumulation of MP in the soil has the potential to interfere with earth system processes such as NPP (Rillig and Lehmann, 2020). Enhanced NPP, i.e., enhanced plant growth, will lead to a change in root exudation quantity and quality. Increased C allocation to roots will likely also alter C allocation to AMF and subsequently process rates of P and N cycling, for which AMF play a major role (van der Heijden et al., 2008). Furthermore, AMF play a role in carbon cycling in the soil (Cheng et al., 2012; Averill et al., 2014; Leifheit et al., 2015). It has been postulated that during the decomposition of labile litter AMF stimulate other microbes that increase decomposition (Cheng et al., 2012) and thus soil respiration, with more C loss as CO_2_. MP-induced changes in AMF activity would thus create a feedback loop to the atmosphere, where increased levels of CO_2_ foster NPP that is again influenced by MP, while at the same time plant productivity is the main resource for AMF determining their activity.

Another earth system feedback loop might occur with nitrogen. Nutrient cycles around the globe are changing, mainly the N cycle due to ubiquitous N deposition. N inputs introduce nutrient imbalances in the soil leading to altered microbial activity. MP induced increase in microbial activity (and mobilization of N) and N inputs could lead to more N_2_O emissions from soil. Increased N release in the soil plus atmospheric N deposition could reduce AMF performance, possibly affecting AMF’s potential to reduce N emissions from soil (Asghari and Cavagnaro, 2012; Storer et al., 2018; Sosa-Hernández et al., 2019), thus fostering greenhouse gas emissions from the soil to the atmosphere. MP thus has the potential to alter the nitrogen cycle and the role of AMF in the cycle, finally leading to increased turnover rates and reduced AMF activity.

## Future Research Priorities

**Figure 1:**
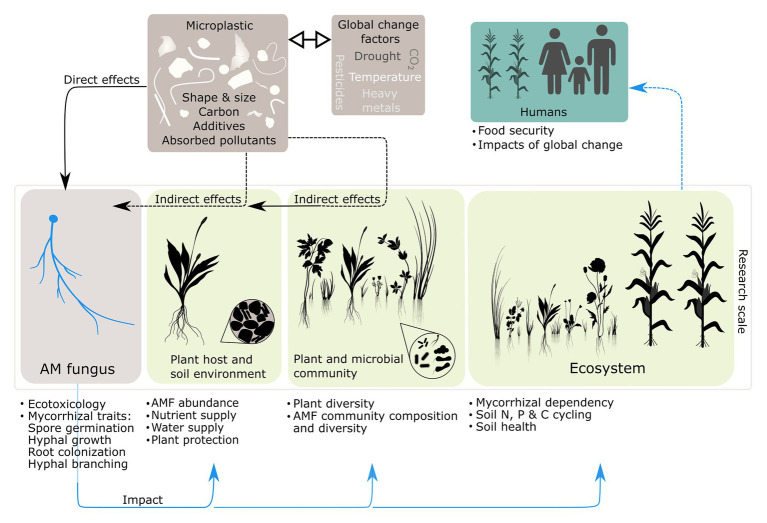
Proposed research scales for microplastic (MP) effects on arbuscular mycorrhizal fungi (AMF) and the corresponding research topics for AMF (text fields). Dashed lines represent indirect effects and solid lines represent direct effects. Black arrows represent the potential impact of microplastic and blue arrows represent the potential impact of microplastic-induced changes in the arbuscular mycorrhiza (AM) symbiosis. The ⇿ symbol indicates potential interactions of microplastic with other factors of global change (GCFs).

At the moment, we cannot fully assess the relevance of MP for AMF. We here propose research areas at individual scales, the corresponding research topics for microplastic effects on AMF and give a conceptual summary of these ideas in Figure 1.

As AMF are obligate symbionts, it will be difficult to disentangle direct impacts of MP on AMF and indirect effects *via* plants, soil properties, or the microbial community. Therefore, basic ecotoxicological research is needed for AMF, how MP affects the AMF community composition, the diversity and their functioning. For these studies, MP with known or without additives is needed, to differentiate between effects from chemistry and plastic traits such as shape or size. In order to draw generalizations for MP effects on this key symbiont, studies should then use different MP (with known chemistry) and soil types. Such experiments with a focus on the fungal part of the symbiosis would need to use compartmentalized designs, in which the fungal extraradical mycelium in the soil is physically separated from the root system, using mesh impenetrable to roots. The addition of MP would then be in these fungal compartments to prioritize effects on the mycobionts. More controlled studies could also be carried out using soil-free *in vitro* culture systems; here, also the fungal mycelium could be separated from the root.

Once this baseline is established, the focus should move to the study of interactions of MP with other GCFs and their effects on AMF. For this question, factorial experiments with other key factors are needed, or new types of designs that can simultaneously take into account a larger number of factors (e.g., Rillig et al., 2019d) should be pursued. Such experiments should focus on the entire plant-AM symbiotic system and its responses.

Research on MP in soils has emerged only a few years ago and is still based on a number of assumptions, because of the lack of systematic MP quantification in soils, in part due to the absence of suitable high-throughput analytical methods. Hence, our knowledge about MP concentrations in the environment is limited. Additionally, concentrations will differ by orders of magnitude according to the distance from a point source of emission. Current laboratory research thus uses concentrations that might not be representative of the situation in the field. Moreover, effects of MP depend on polymer type, its additives and shape (Rillig et al., 2019c), and current analytical methods do not capture this level of detail. Due to these issues (uncertain concentration and chemistry), it is difficult to perform observational studies in the field with native AMF communities under realistic conditions.

Finally, research also needs to move to the plant community level, to more fully explore how AMF and a host community respond to MP. This type of experiment also opens the door to an ecosystem-level assessment (Rillig and Lehmann, 2020), and could include treatments in which mycorrhizal fungi have either been added or not; this way, we will learn how a plant community-level or ecosystem-level response to MP will depend on mycorrhiza.

## Data Availability Statement

The original contributions presented in the study are included in the article/supplementary material, further inquiries can be directed to the corresponding author.

## Author Contributions

EL wrote the first draft of the paper. EL and AL created the artwork. All authors contributed to the article and approved the submitted version.

### Conflict of Interest

The authors declare that the research was conducted in the absence of any commercial or financial relationships that could be construed as a potential conflict of interest.
